# Supplementary figures and images for: The Community Assessment of Psychic Experiences-Positive scale (CAPE-P15) accurately classifies and differentiates psychotic experience levels in adolescents from the general population
Source: PLoS One. 2021 Aug 26;16(8):e0256686. doi: 10.1371/journal.pone.0256686 (PMC8389461; doi:10.1371/journal.pone.0256686)

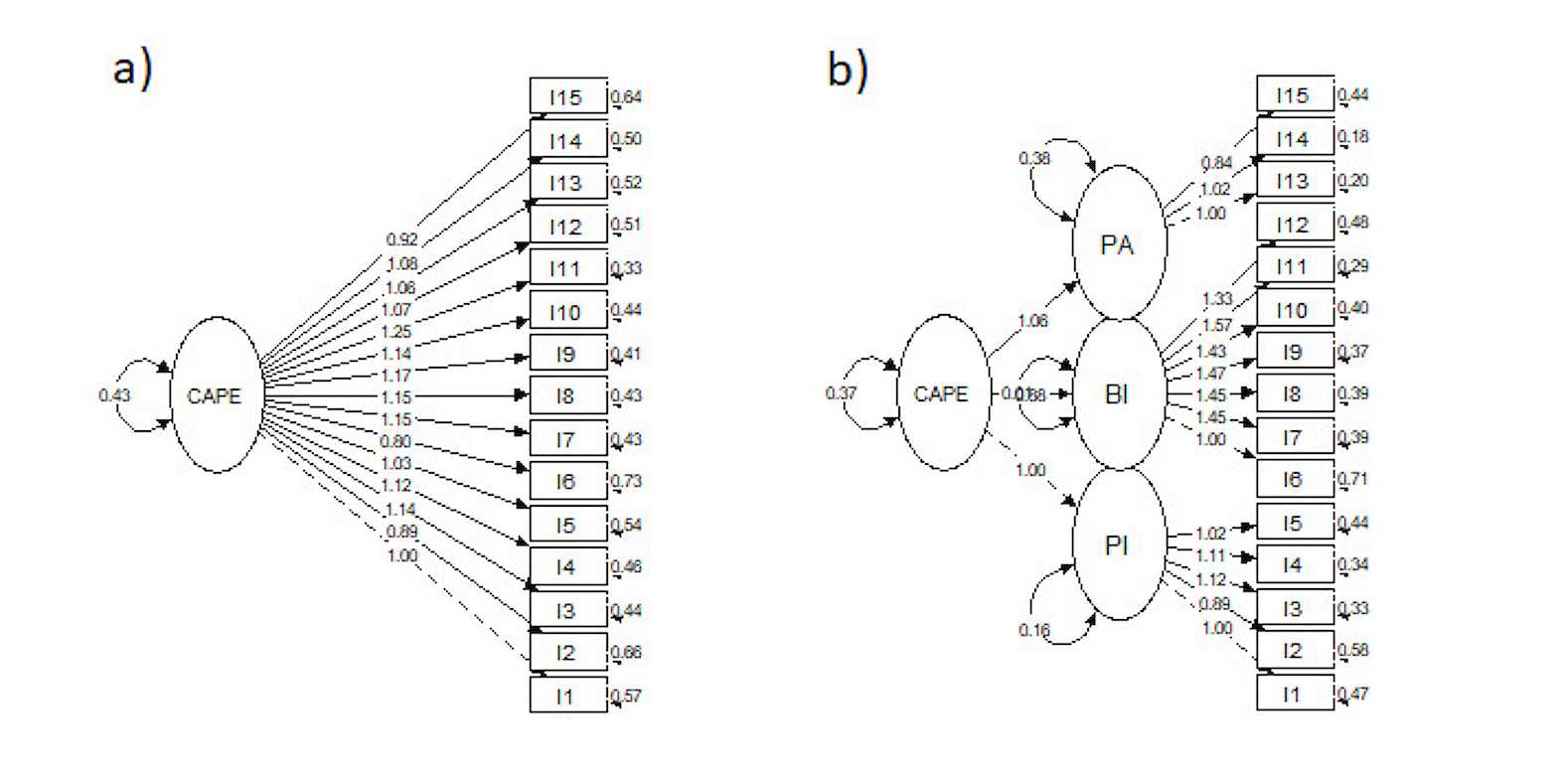

Supplement: S1 Fig — A) General factor mode. B) Hierarchical model. (TIF) [file pone.0256686.s001.tif]
